# Can carbon monoxide-poisoned victims be organ donors?

**DOI:** 10.1186/2045-9912-4-13

**Published:** 2014-07-31

**Authors:** Noritomo Fujisaki, Atsunori Nakao, Takaaki Osako, Takeshi Nishimura, Taihei Yamada, Keisuke Kohama, Hiroyuki Sakata, Michiko Ishikawa-Aoyama, Joji Kotani

**Affiliations:** 1 Department of Emergency, Disaster and Critical Care Medicine, Hyogo College of Medicine, Nishinomiya, Hyogo, Japan

## Abstract

The increasing demand for organ allografts to treat end-stage organ failure has driven changes in traditional donor criteria. Patients who have succumbed to carbon monoxide (CO) poisoning, a common cause of toxicological mortality, are usually rejected as organ donors. To fulfill the increasing demand, selection criteria must be expanded to include CO-poisoned donors. However, the use of allografts exposed to high CO concentrations is still under debate. Basic research and literature review data suggest that patients with brain death caused by CO poisoning should be considered appropriate organ donors. Accepting organs from CO-poisoned victims could increase the number of potential donors and lower the death rate of patients on the waiting lists. This review and reported cases may increase awareness among emergency department physicians, as well as transplant teams, that patients dying of CO exposure may be acceptable organ donors.

## Introduction

The success of organ transplantation has led to a growing imbalance between the supply and demand of donor organs. Accordingly, the use of organs from nonconventional donors, such as those who die from carbon monoxide (CO) poisoning, has increased. Organ procurement is based on criteria that exclude as potential donors those who have undergone prolonged cardiopulmonary resuscitation (CPR), those with certain metabolic diseases, and those with infectious diseases such as human immunodeficiency virus (HIV), hepatitis, sepsis, tuberculosis, and metastatic disease. These exclusion criteria result in a donor shortage despite an increasing number of individuals waiting for organ transplants.

CO is well-known in clinical medicine for its toxicity, which is due to its ability to interfere with oxygen delivery. Traditionally, CO poisoning has been considered a contraindication to organ procurement. The possibility of organ procurement from donors who died of CO poisoning has been discussed in the scientific literature. The possibility of permanent and significant injury to transplanted organs caused by CO may raise serious concerns regarding the procedure. However, a number of publications report successful organ transplantation from donors who were brain dead as a result of CO poisoning. Therefore, the belief that this poisoning automatically contraindicates donation of organs, including the heart, kidney, liver, may be unfounded.

CO has recently raised scientific and clinical interest, as its beneficial effects and mechanisms of action have been substantially defined in various *in vitro* and *in vivo* studies [1]. Although CO is generally known as a toxic gas, it is endogenously produced in the body and functions as an important signaling gas molecule, providing potent cytoprotective effects [2,3]. These research findings may support the idea that CO-poisoned donors should be considered appropriate for organ transplantation.

The use of organs from CO-poisoned victims for the purpose of transplantation has been poorly studied; criteria for organ donation are virtually non-existent in such cases [4,5]. The aim of this review is to provide an outline of previous reports of transplants from CO-poisoned donors, as well as current knowledge from CO-related clinical studies. In particular, emergency physicians must be aware that patients dying from CO exposure may be acceptable organ donors, because the emergency department is a critical site for organ procurement.

### CO poisoning and organ injury

While CO poisoning is common, its incidence is uncertain and frequently unrecognized, as the signs and symptoms are relatively nonspecific [6]. Unfortunately, no marker or constellation of signs or symptoms at presentation predict long-term outcome following CO poisoning [7,8]. Given the neurocognitive sequelae following CO poisoning, increased awareness and prevention of CO poisoning is imperative [9].

CO has a 200 times greater chemical affinity for hemoglobin than oxygen. The mechanisms of tissue damage due to CO poisoning are as follows: oxygen delivery to the tissues is reduced due to the high chemical affinity of CO to hemoglobin, resulting in the shift of the oxyhemoglobin dissociation curve to the left, and CO strongly binds to myoglobin, interfering with oxygen transportation to the mitochondria.

Various studies have demonstrated that CO interferes with myoglobin, P450, and other enzyme functions; causes lipid peroxidation through neutrophil activation; produces oxidative stress manifested by peroxynitrate deposition in endothelium; binds to cytochrome A3, disrupting intracellular oxygen utilization; can cause neuroexcitotoxicity; and contributes to hippocampal cellular death through apoptosis [10]. The tissues most affected are those most sensitive to oxygen deprivation, specifically those of the central nervous system and myocardium. Individual susceptibility varies and symptoms depend on degree of exposure (concentration, duration, and ventilation volume), tissue metabolic demands, concurrent anemia, and preexisting atherosclerosis.

Usually patients with CO poisoning also suffer from burns. Of note, admission to the hospital after a fire accident may have been exposed the patient to cyanide gases as well as CO [11].

A comparison between successful and unsuccessful transplantation cases with CO-poisoned donor organs would be quite interesting. Identifying some key characteristics of CO-poisoned donors would be beneficial for future application. However, unfortunately, most of the reports were single case reports or poorly described without detailed data, including data on COHb levels, CO poisoning time, and clinical course.

### Donor treatment with CO: basic research data

CO potently protects against cellular injury. CO relaxes blood vessels and exerts anti-thrombotic effects by inhibiting platelet aggregation and derepressing fibrinolysis. In addition, CO reduces ischemia/reperfusion injury and inflammatory responses. CO inhibits apoptosis of endothelial and epithelial cells and reduces proliferation of smooth muscle cells, fibroblasts, and T lymphocytes. Thus, accumulating evidence supports the notion that CO treatment of transplant donors, organs, or recipients can prevent graft dysfunction due to rejection or ischemia/reperfusion injury.

Several studies have demonstrated that donor pretreatment with CO benefits graft function in animal models. In an islet allograft model, donor CO pretreatment blocked toll-like receptor-4 upregulation, diminishing the inflammatory response and cytokine-induced apoptosis, which protected the graft from rejection [12]. Donors who have inhaled CO or suffered cold ischemia with CO perfusion have demonstrated improved graft function, which was associated with decreased apoptosis and increased viability of endothelial cells and cardiomyocytes [13]. Induction of CO in the donor by oral administration of methylene chloride was able to prevent chronic rejection of rat renal allografts [14]. Donors treated with CORM-2 presented fewer lymphocytic infiltrates and reduced acute tubular necrosis in the graft [15]. This protection was most likely related to CO-induced endothelial changes via a reduction in NADPH-dependent superoxide anion production, IkB degradation, and E-selectin and ICAM-1 expression [15]. Studies on CO’s mechanism of action have shown that it binds to the heme moiety of soluble guanylyl cyclase (sGC), leading to cyclic guanosine monophosphate (cGMP) activation [16].

As shown above, donor treatment with CO was beneficial for the survival of several types of organ grafts such as the heart, kidney, liver, and lung in animal models; we might anticipate similar efficacies of CO in grafts harvested from CO-poisoned donors in humans.

### Clinical reports

Several studies report the successful use of CO-poisoned victims as organ allograft donors (Table 1). Bojakowski *et al*. reported a single case of renal transplantation with grafts retrieved from a CO-poisoned donor. Despite prolonged warm ischemic time (>100 minutes), no complications were observed in the post-transplant course, suggesting that CO diminished ischemia/reperfusion injury and rejection [17]. Luckraz *et al*. described their experience with seven patients who were transplanted using organs after fatal CO poisoning (six heart transplants and one single lung transplant). History of CO inhalation was obtained in all of these donors. Five of the six heart transplant patients were alive and well at the time of the study, with survival ranging from 68 to 1,879 days. One patient (a 29-year-old male) died 12 hours post-transplant due to donor organ failure. The patient who had a right single lung transplant did well initially after the transplant, but died after eight months due to *Pneumocystis carinii* pneumonia. All recipients who were transplanted from CO-poisoned donors and ventilated for more than 36 hours survived for more than 30 days [18].

**Table 1:** List of the past report using CO-poisoned victims as organ allograft donors

**Year**	**Author**	**Journal**	**# of donors**	**Reference**
1989	Karwande, et el.	J Heart Transplant	1 heart	[19]
1992	Hebert MJ, et al.	N Engl J Med	6 kidneys	[20]
1992	Shennib H et al.	J Heart Lung Transplant	1 lung	[21]
1992	Smith JA, et al.	J Heart Lung Transplant	2 hearts	[22]
1993	Iberer F, et al.	J Heart Lung Transplant	1 heart	[23]
1994	Leikin JB, et al.	Am J Emerg Med	10 kidneys, 3 livers	[4]
1995	Roberts JR et al.	Ann Emerg Med	1 heart	[24]
1995	Hantson P, et al.	J Toxicol Clin Toxicol	1 kidney	[25]
1996	Verran D, et al.	Transplantation	2 livers	[26]
1997	Koerner MM, et al.	Transplantation	5 hearts	[27]
2001	Rodrigus IE, et al.	J Heart Lung Transplant	1 heart	[28]
2001	Bentley MJ, et al.	Ann Thorac Surg	2 hearts, 2 kidneys, 1 pancreas	[29]
2001	Luckraz H, et al.	Ann Thorac Surg	6 hearts, 1 lung	[18]
2007	Bojakowski K, et al.	Transplant Proc	1 kidney	[17]
2008	Sezgin A, et al.	Transplant Proc	1 heart	[30]
2008	Martin-Suarez S, et al.	Transplant Proc	1 heart	[31]

Koerner *et al*. reported five cardiac allografts from brain-dead, CO-poisoned donors. Donor history showed CO intoxication in all cases. At the time of organ explantation, donor hemodynamic parameters were weak in all patients. The postoperative course was uneventful in three of the five recipients. The overall three-year survival rate in this small group was 40%. Induction therapy or rescue therapy with mono/polyclonal antibodies was not necessary. Myocardial right-ventricular biopsies did not show any specific signs of CO-poisoning [27].

Verran *et al*. reported two successful liver transplant cases using CO-poisoned donors. There was satisfactory early function of both allografts, although marked patchy necrosis was seen on the postreperfusion biopsy (case 1) and on a 10 day postoperative biopsy (case 2). In both cases, the changes were considered to be related to damage sustained from CO inhalation. Both allografts soon achieved normal function and both recipients were well at the time of the study. They concluded that CO-poisoning can cause liver damage that can recover completely following liver transplantation. Shennib *et al*. reported a single case of successful lung transplantation using a CO-poisoned donor [21]. Sezgin *et al*. presented a patient who underwent a successful cardiac transplantation from a brain-dead donor who had CPR after CO intoxication. Although the donor had a history of CPR, the left ventricular ejection fraction was 55% and the echocardiographic evaluation revealed normal cardiac contractions with acceptable hemodynamic parameters. While positive inotropic support was needed in the early postoperative period, any changes related to intoxication in the endomyocardial biopsy was not observed [30].

On the other hand, contradictory evidence concerning this topic has also been published. Karwande *et al*. presented a case of CO poisoning that led to severe myocardial damage in the transplanted heart [19]. Hantson *et al*. reported one case of kidney transplantation from a CO-poisoned donor. In this case, the recipient was extremely unstable and the graft was rejected within a few months [25]. Rodrigus *et al*. presented a case of CO poisoning in a multiorgan donor that led to primary cardiac allograft failure. A biventricular assist device was used as a bridge to recovery [28].

Considering the significant risk of donor organ failure, a cautious approach is still warranted. A formal approach of invasive monitoring and active management further improves the chances of successful outcome. Predicting organ damage by blood carboxy-hemoglobin levels is quite challenging because very poor correlation has been found between blood carboxy-hemoglobin level, tissue carboxy-hemoglobin level, and degree of organ damage [32].

In general, little data regarding long-term survival are available, but individual reported cases suggest that the survival of patients transplanted with organs procured from carefully selected CO-poisoned donors may be comparable to that of patients transplanted from non-poisoned donors. While these reports do not support the use of CO therapeutically during transplantation *per se*, they certainly do not provide any evidence that contraindicates it.

## Conclusion

Currently, it is estimated that less than 1% of all donated organs in Western Europe and the USA are procured from CO-poisoned donors [33]. Although reports of successful solid organ transplantation using organs from CO-poisoned donors have been published, there have also been reports of donor organ failure, resulting in early recipient death. Thus, the use of these organs for transplantation remains controversial, as little data exists from which to draw any firm conclusion. Since CO poisoning is common, organs procured from CO-poisoned donors could save the lives of those on donor waiting lists.

## Competing interests

The authors declare that they have no competing interests.

## Authors’ contribution

NF and AN are responsible for writing the manuscript. TO, TN, TY, KK, HS, MIA and JK are responsible for drafting and revision. All authors read and approved the final manuscript.
